# Properties of acid‐induced currents in mouse dorsal root ganglia neurons

**DOI:** 10.14814/phy2.12795

**Published:** 2016-05-12

**Authors:** Zuhal Ergonul, Lei Yang, Lawrence G. Palmer

**Affiliations:** ^1^ Department of Physiology and BiophysicsWeill Cornell Medical CollegeNew YorkNew York; ^2^ Department of PediatricsNewYork‐Presbyterian/Weill Cornell Medical CenterNew YorkNew York; ^3^ Department of PhysiologyHarbin Medical UniversityHarbinChina

**Keywords:** Amiloride, ASIC, Pc1Tx, sustained currents, Zn^2+^

## Abstract

Acid‐sensing ion channels (ASICs) are cation channels that are activated by protons (H^+^). They are expressed in neurons throughout the nervous system and may play important roles in several neurologic disorders including inflammation, cerebral ischemia, seizures, neurodegeneration, anxiety, depression, and migraine. ASICs generally produce transient currents that desensitize in response to a decrease in extracellular pH. Under certain conditions, the inactivation of ASICs can be incomplete and allow them to produce sustained currents. Here, we characterize the properties of both transient and sustained acid‐induced currents in cultured mouse dorsal root ganglia (DRG) neurons. At pH levels between 7.3 and 7.1 they include “window currents” through ASICs. With stronger acid signals sustained currents are maintained in the absence of extracellular Na^+^ or the presence of the ASIC blockers amiloride and Psalmotoxin‐1(PcTx1). These sustained responses may have several different origins in these cells, including acid‐induced stimulation of inward Cl^−^ currents, block of outward K^+^ currents, and augmentation of inward H^+^ currents, properties that distinguish these novel sustained currents from the well‐characterized transient currents.

## Introduction

The acid‐sensing ion channels (ASICs) are cation channels that are activated by protons (H^+^) and are expressed in neurons throughout the nervous system. ASICs are part of a superfamily of channels that includes the epithelial Na channel (ENaC), FMRFamide‐gated channels (FaNaC), and mechanosensitive channels in the MEC/DEG family (Grunder and Pusch 2015; Kellenberger and Schild 2015). They are weakly voltage‐dependent and have a variable selectivity for Na^+^ over K^+^ and other cations (Yang and Palmer 2014; Grunder and Pusch 2015).

Since the first reports of proton‐induced depolarizing sodium currents in sensory neurons from Krishtal and colleagues in 1981 (Krishtal and Pidoplichko 1981), a growing body of evidence has accumulated showing the important role of ASIC channels in nociception (Deval and Lingueglia 2015; Krishtal 2015). It now appears that ASICs can sense synaptically released protons (H^+^) as well as sustained acidosis during various pathophysiological states (Grunder and Pusch 2015). ASICs are primarily permeable to Na^+^ and elicit cell depolarization, resulting in signaling through the neurons expressing them. Prolonged activation of the channels may lead to secondary intracellular accumulation of calcium (Ca^2+^) and neurotoxicity. As a result, their activation may be involved in neuronal aciditoxicity, a process demonstrated to play important roles in several neurologic disorders including inflammation, cerebral ischemia, seizures, neurodegeneration, anxiety, depression, and migraine. Therefore, ASICs represent novel targets for potential treatments of these disorders (Sherwood et al. 2012; Wemmie et al. 2013; Benarroch 2014).

ASICs generally produce transient currents that desensitize in response to a decrease in extracellular pH. Under certain conditions, the inactivation of ASICs can be incomplete and allow them to produce sustained currents in the continued presence of H^+^ (Lingueglia et al. 1997; Waldmann et al. 1997b). A sustained component of H^+^‐induced current has been suggested as the underlying mechanism to sense acidosis. These currents have been ascribed to activation of ASIC3 subunits that are thought to mediate the nonadaptive pain caused by sustained acidosis (Salinas et al. 2009).

Several amino acids have proton‐binding affinities in the physiological range and pH affects the properties of most proteins. Sustained currents at extremely low pH may represent specific biophysical gating properties of ASICs, but multiple other structures and ions might also be involved (Krishtal 2015), and caution in interpretation of these acid‐induced currents is necessary (Yagi et al. 2006).

Sustained currents induced by extreme acidosis are insensitive to ASIC blockers. For example, APETx2, a specific blocker of ASIC3, is ineffective against sustained currents (Diochot et al. 2004). The nonspecific ASICs blocker amiloride and the gating modifier Psalmotoxin1 (PcTx1), a specific blocker for ASIC1a, paradoxically activate sustained currents in both transfected cells and DRG neurons (Yagi et al. 2006; Grunder and Pusch 2015). Furthermore, for some family members of ASICs, the sustained current is unselective, whereas the transient current is Na^+^‐selective (Lingueglia et al. 1997; Springauf and Grunder 2010).

There are relatively few systematic studies on sustained acid‐induced currents, and a better understanding of them would enhance our perspectives for ASIC‐induced nociception and acidotoxicity. Here, we explore the impact of different ions on acid‐sensing sustained currents in cultured mice dorsal root ganglia (DRG) neurons, and the paradoxical activating effects of amiloride and PcTx1. Our results suggest that sustained currents may have several different origins in these cells.

## Methods

### Animals

All procedures using animals were approved by the Institutional Animal Care and Use Committee of Weill Cornell Medical College. Swiss Webster mice (Charles River Laboratories, Kingston, NY) from postnatal day 0 through postnatal day 2 were used for the preparation of DRG neurons.

### Primary culture of DRG neurons

Cultures of dorsal root ganglia (DRG) neurons were prepared as described previously with minor modifications (Lindsay 1988; Mannsfeldt et al. 1999; Chakrabortty et al. 2000; Leng et al. 2013) (Lindsay 1988). Mouse pups were sedated by carbon dioxide inhalation and decapitated. DRGs were isolated from all lumbar spinal levels and freed from connective tissue in Leibovitz's L‐15 medium (Gibco, Gaithersburg, MD). DRGs were subsequently incubated in 0.05% trypsin (Invitrogen, Carlsbad, CA) for 20 min at 37°C. Trypsin was removed by washing with dissociation medium DMEM (Life Technologies GmbH, Darmstadt, Germany) containing 10% heat‐inactivated horse serum (Biochrom, Cambridge, UK), 100 U penicillin and 100 mg/mL streptomycin (Life Technologies). DRGs were dissociated into single cells by triturating with a fire‐polished pasteur pipette. They were cultured in Neurobasal medium supplemented with 1X B‐27, 100 ng/mL NGF, 2 mmol/L glutamine, 20 *μ*mol/L 5‐fluorodeoxyuridine, 100 IU/mL penicillin and 100 *μ*g/mL streptomycin. Cells were plated on poly‐l‐lysine (200 mg/mL) and laminin (20 *μ*g/mL) coated plastic coverslips and kept at 37°C in 5% CO_2_. Large to mid‐size multipolar and bipolar neurons, 12–17 microns in diameter, were used for electrophysiological recordings 2–8 days after plating. These were selected because they tolerated extended recordings under whole‐cell conditions.

### Electrophysiology

Patch‐clamp pipettes were prepared from hematocrit capillary glass (VWR Scientific, Radnor, PA) using a vertical puller (David Kopf Instrument, 700C) modified to pull in three stages. They had resistances of 5–8 MΩ. The pipette solution contained (in mmol/L): 140 KCl, 11 EGTA, 2 MgCl_2_, 1CaCl_2,_ 10 NaCl, 2 MgATP, and 10 HEPES, with pH adjusted to 7.4 with N‐methyl‐D‐glucamine (NMDG). The bath solution contained (in mmol/L): 135 NaCl, 2 CaCl_2_, 1 MgCl_2_, 5 KCl, 10 glucose, and 10 HEPES with pH adjusted to various values with NMDG. Modifications to these basic solutions are described in the text. Changes in extracellular K were made by substitution with Na. Reductions in pipette Cl^−^ were made by substitution with aspartate. Solutions were rapidly changed during recordings using gravity‐fed flow pipes positioned near the cell. Whole‐cell currents were recorded with an EPC‐7 patch‐clamp amplifier (HEKA) and digitized with a Digidata 1332A interface (Molecular Devices, Sunnyvale, CA). We did not use series‐resistance compensation as in the absence of activation of voltage‐gated channels the input resistance of the cell (~500 MΩ) was much larger than the pipette resistance. All recordings were performed at room temperature. Data were analyzed using Clampfit software (Molecular Devices).

## Results

### Responses to acidification

**Figure 1:**
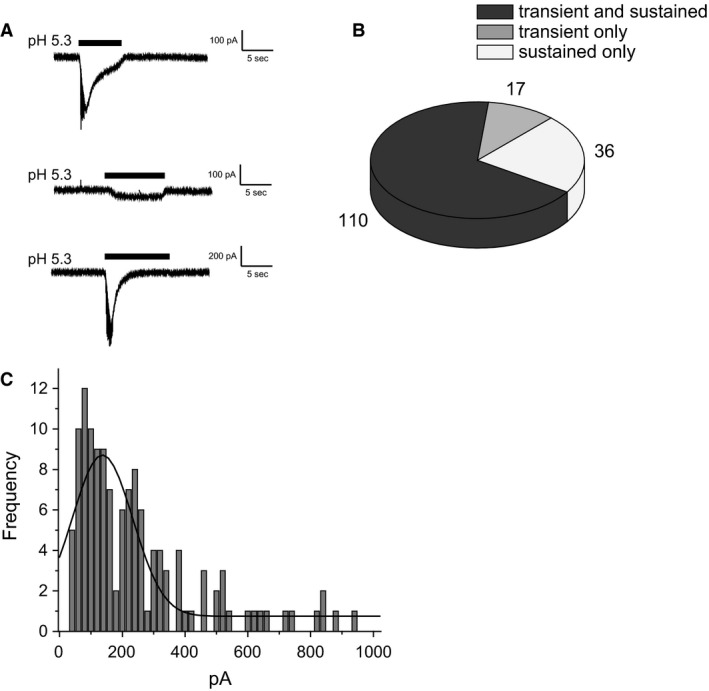
Proton‐evoked (pH 5.3) currents in mouse DRG neurons. (A) examples of transient plus sustained currents (top), sustained‐only currents (middle), and transient‐only currents (bottom). (B) numbers of neurons responding to low pH with patterns illustrated in A with pH ≤ 6.3. (C) Frequency histogram of peak amplitude of transient currents. The line represents the best‐fit to a Gaussian distribution with mean = 137 pA and standard deviation = 96 pA.

We studied mid‐size to large multipolar and bipolar neurons which comprised approximately 10–20% of the cells. Cultured cells started to show acid sensitivity after 48 h of plating. We did not use cells after 8 days as they became fragile and whole‐cell currents were unstable. The cells responded to a rapid change in bath solution from pH 7.4 to ≤ 7.2 with one of three patterns (Fig. 1A). The first consists of a fast transient inward current that fully inactivates, whereas the pH remains acidic. This response often triggered a series of current spikes at the beginning of the low pH solution change, presumably resulting from action potentials elicited in the poorly clamped processes of some neurons. These transient‐only currents were best observed at mild acidification and were very rare at pH < 6.0. The second pattern was a fast transient current with a sustained component that does not fully inactivate if the pH remains acidic. These currents were also able to trigger action potentials in some neurons. Third, more severe acidifications with pH ≤ 6.0 produced a sustained inward current in some cells without generating any transient component. These sustained currents were not associated with action potentials and were rare at pH > 6.5.

Figure 1 shows the three different types of proton‐evoked currents and summarizes the percentage of neurons with those currents, and frequency of peak amplitude with a pH challenge to ≤ 6.3. Experiments using external pH between 5.3 and 6.3 gave similar results and were therefore pooled. Most neurons generated “ASIC‐like” transient currents with a sustained component (67%). A smaller percentage showed transient‐only currents (10%), whereas 23% had sustained currents without any transient component. In most cells the transient currents had peak amplitudes between 50 and 250 pA The frequency of peak amplitudes of these currents followed a Gaussian distribution with a peak at ~ 140 pA. This distribution is consistent with a single population of cells. Those that lacked a transient response would comprise a second population.

**Figure 2:**
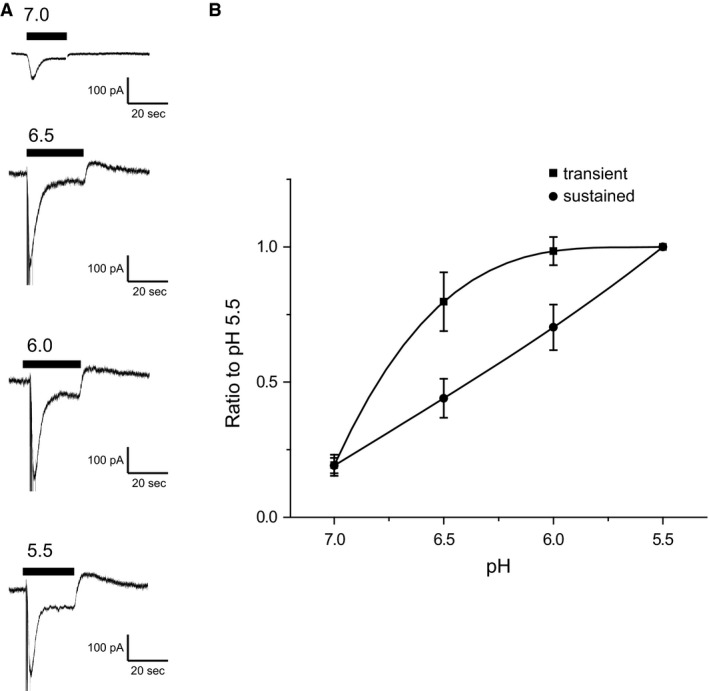
pH‐dependent activation of transient and sustained currents in mouse DRG neurons. (A) Recordings from neurons in response to a low pH challenge (responding to steps from 7.4 to 7.0, 6.5, 6.0, and 5.5, respectively). (B) pH‐dependent activation of transient currents and sustained currents. Data are normalized to values obtained at pH 5.5 and are represented as means ± SEM for four recordings. The pH required for a half‐maximal response (pH_50_) was 6.8 for transient currents. Sustained currents did not exhibit a maximal amplitude.

We next compared the pH dependence of transient and sustained currents. Figure 2 shows currents as a function of pH, normalized to values at pH 5.5. The transient currents exhibited saturation as pH was reduced with a half‐maximal response at ~pH 6.8. In contrast, sustained currents continued to increase as the pH was lowered to 5.5.

**Figure 3:**
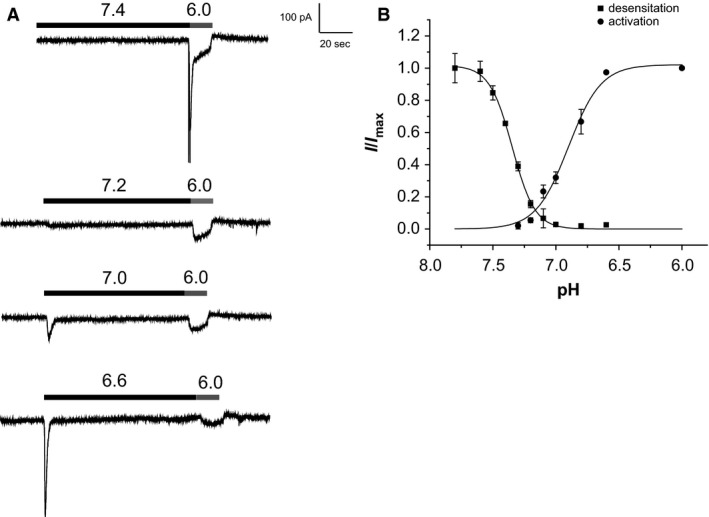
Activation and desensitization curves of proton‐evoked transient currents. (A) Conditioning steps of different pH between 7.4 and 6.6 are followed by a test step with pH 6.0. (B) Peak values of currents at pH 6.0 were used for the desensitization curve, and peak currents during the conditioning steps were used for the activation curve. Currents were normalized to those measured with pH 7.8 (desensitization) and pH 6.0 (activation), and are represented as mean ± SEM for seven cells (activation) and 21 cells (desensitization). Solid lines represent best fits of the Hill equation with half‐activation at pH 6.9, and half‐desensitization at pH 7.35.

In order to further compare transient and sustained currents, we generated activation and desensitization profiles using conditioning steps to various pH values followed by a standard challenge with pH 6.0. Peak currents during the conditioning steps were used for the activation curve and peak currents at pH 6.0 were used to generate the desensitization curve (Fig. 3B). Activation curves were half‐maximal at about pH 6.9, whereas desensitization curves were half‐maximal between 7.3 and 7.4. This leaves a measurable window current around pH 7.2. These currents were quite small (<5%) relative to peak currents but were evoked over a patho‐physiological pH range.

### Ionic basis of sustained currents

**Figure 4:**
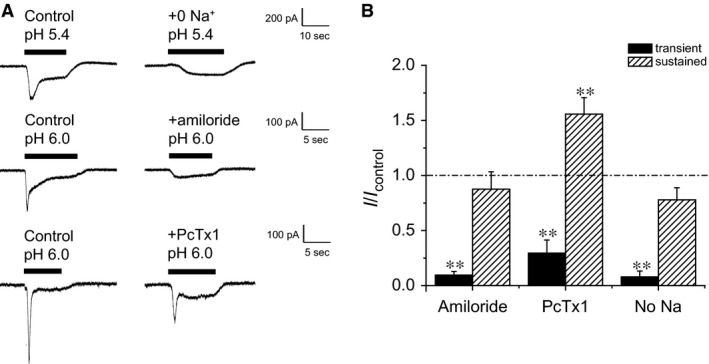
Effects of amiloride (1 mmol/L), PcTx1 (20 nmol/L) and Na^+^‐free extracellular solution on proton‐evoked transient currents and sustained currents in mouse DRG neurons. Amiloride and PcTx1 were added only to the low‐pH solution. (A) Recordings from neurons in response to a low pH challenge and inhibition of transient currents. (B) Values of peak and sustained currents normalized to control values. Data are represented as means ± SEM for 5–6 cells. Transient currents were significantly decreased with amiloride, PcTx1, and removal of Na^+^ from extracellular solution. Amiloride and removal of Na^+^ had no effects on sustained currents, whereas PcTx1 increased them **P < 0.01 compared with control).

ASICs are cation channels that select for Na^+^ over other cations. We therefore explored the effects of Na^+^ ion removal on both transient and sustained currents. Replacing Na^+^ in extracellular solutions with NMDG^+^, a large cation that we presume neither permeates nor blocks the channels, abolished transient currents but did not change sustained currents (Fig. 4A). Then, we tested the effects of the ASIC blockers amiloride (Sigma‐Aldrich, St. Louis, MO) and PcTx1 (Abcam Inc., Cambridge, MA) on both currents. These compounds reduced or abolished transient currents, but did not inhibit sustained currents. In fact, PcTx1 increased the magnitude of the sustained response.

**Figure 5:**
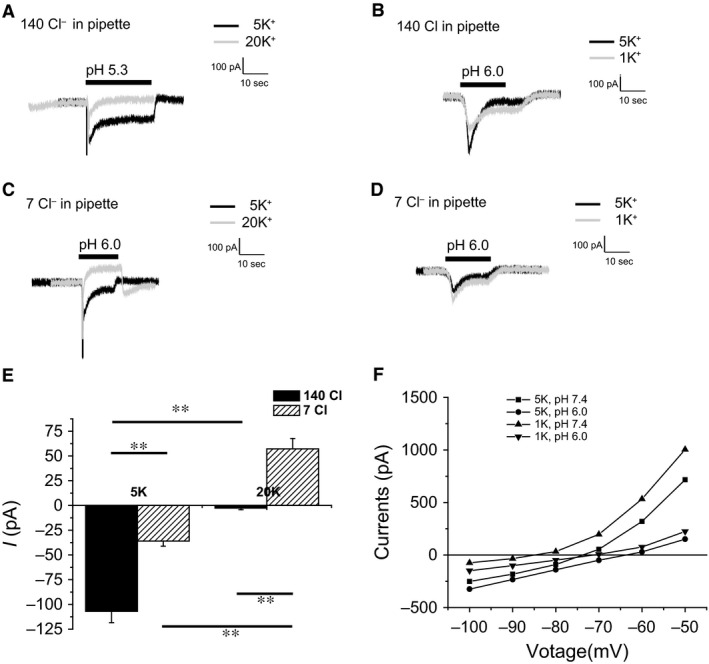
Effects of extracellular K^+^ and intracellular Cl^−^ concentrations on proton‐evoked currents. (A) Reduction in sustained current with extracellular high K^+^. (B) Increased sustained current with low extracellular K^+^. (C) Reversal of sustained current with increased extracellular K^+^ in the presence of 7 mmol/L intracellular Cl. (D) Increase in sustained currents with reduced extracellular K^+^. in the presence of 7 mmol/L (E) Values of sustained currents with high (140 mmol/L) and low (7 mmol/L) intracellular Cl‐ and high (20 mmol/L) and low (5 mmol/L) extracellular K^+^. Data are represented as means ± SEM for 3–11 cells. (*P < 0.05, **P < 0.01) (F) Comparison of I‐V curves at normal and low extracellular K^+^ conditions. Results are from a single cell, representative of five independent experiments (1 mmol/L (K) and six experiments (5 mmol/L K).

Since sustained currents were observed in the absence of Na^+^, we decided to explore the involvement of other ions including K^+^, Cl^−^, and H^+^ which may contribute to the generation of these responses. In order to test for K^+^ currents, we changed the driving force for K^+^ by increasing (to 20 mmol/L) or decreasing (to 1 mmol/L) its concentration in the bath solutions, exchanging K^+^ for Na^+^. Sustained currents decreased with 20 mmol/L K^+^ (Fig. 5A) and increased with 1 mmol/L K^+^ (Fig. 5B) consistent with the idea that they could reflect in part blockade of outward K^+^ currents by protons. We also reduced the Cl^−^ concentration of the pipette solutions to 7 mmol/L (instead of 140 mmol/L) by substitution with aspartate. This reduced the sustained acid‐induced currents recorded with 5 mmol/L K^+^ and reversed the currents in the presence of 20 mmol/L K^+^ (Fig. 5C, D and E). The simplest interpretation is that low pH induces an outward flow of Cl^−^ and independently blocks an inward flow of K^+^.

Comparison of I‐V curves at pH 7.4 and pH 6.0 between regular 5 mmol/L K^+^ bath, and 1 mmol/L low K^+^ bath is shown in Fig. 5F. The large pH sensitive currents at V_m_ > −80 mV probably reflect inhibition of outward K^+^ movement, whereas the smaller acid‐induced currents at V_m_ < −80 mV could include contributions of Cl^−^ and H^+^ fluxes.

### Effects of Zn^2+^

**Figure 6:**
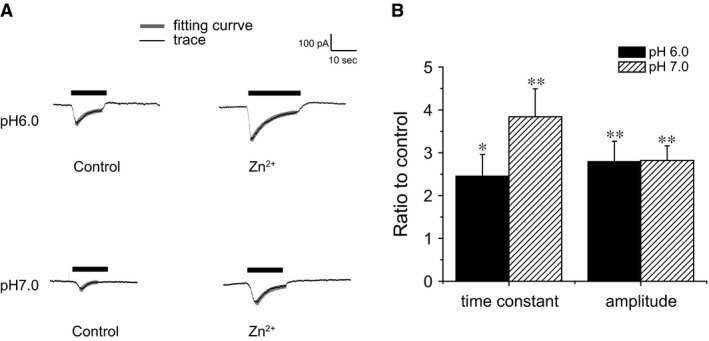
Effects of Zn^2+^ on activation of transient currents. (A) Currents activated by different pH with or without Zn^2+^. Thick gray lines represent best fits to exponential decay functions. (B) Time constants and maximum transient currents from experiments like those shown in panel A. Values are normalized to those of control traces. Data are represented as means ± SEM for seven cells (*P < 0.05, **P < 0.01 compared with control).

With 7 mmol/L Cl^−^ in the pipette and 5 mmol/L K^+^ in the bath the equilibrium potentials for both ions are close to the test potential (−80 mV). This suggests other ions contribute to these currents. One possibility is that they represent inward H^+^ currents. Since protons cannot be removed during the acid challenge we could not test this idea directly. As some H^+^ channels are blocked by extracellular Zn^2+^ (DeCoursey 2013), we examined the effects of this divalent cation on acid‐induced currents in cultured DRG neurons. As shown in Figure 6, although 1 mmol/L Zn^2+^ did not inhibit the sustained responses to low pH, it enhanced the transient responses as described previously for ASIC2a (Baron et al. 2001). This enhancement was due at least in part to a reduced rate of desensitization. As shown in Figure 6, in the presence of Zn^2+^ the time constant for desensitization increased by 2.5‐ to 4‐fold, comparable to the increase in peak current. A similar result was observed for ASIC3 channels (Yagi et al. 2006).

**Figure 7:**
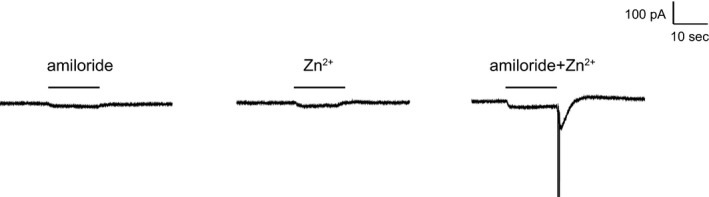
Activation of sustained currents by Zn^2+^ and amiloride, and amiloride‐washoff effect at pH 7.4. (A) Effect of 1 mmol/L amiloride. (B) Effect of 1 mmol/L Zn^2+^. C. Effects of amiloride + Zn^2+^. Simultaneous washoff of amiloride and Zn^2+^ produced a transient inward current. Results are from a single cell, representative of three independent experiments.

Zn^2+^ can bind to ASICs channels and induce currents without a pH change (Baron et al. 2001). As shown in Figure 7, both Zn^2+^ and amiloride elicited small but measurable sustained inward currents in cultured DRG neurons. When the two were added together, their simultaneous removal produced a much larger transient inward current which was accompanied by action potentials. One interpretation is that amiloride activates the channels in a pH‐insensitive manner, but also blocks them. In the presence of Zn^2+^ the channels activate but do not fully desensitize, so that rapid removal of the drug transiently reveals the active state.

## Discussion

Several lines of evidence indicate that ASIC channels are involved in pain pathways in both the peripheral and the central nervous system. Among the different ASIC channels, ASIC1 and ASIC3 display the highest sensitivity to extracellular protons, with activation thresholds just below the physiological pH, around pH 7.0 and pH 7.2, respectively. Activation of ASIC channels containing ASIC1 and/or ASIC3 subunits has a direct impact on the sensory neuron's activity, by generating sufficient depolarization to reach the threshold for action potential triggering, or to sensitize neurons to other stimuli (Deval and Lingueglia 2015). In mouse, ASIC3 has been identified in several different specialized sensory nerve endings of the skin suggesting a role in mechanosensation, in addition to acid‐evoked nociception (Price et al. 2001). Similarly in rat, both small and large mouse DRG neurons, including those innervating muscle tissues, express ASIC3 channels (Sluka et al. 2003).

ASICs may play a role in dural‐afferent signaling as a result of decreased pH in contributing to migraine pain. Amiloride was shown to block cortical spreading depression, the experimental correlate of migraine aura, and inhibited trigeminal activation in in vivo migraine models, via an ASIC1‐dependent mechanism (Holland et al. 2012). In that same study, amiloride also demonstrated good clinical efficacy in a small open‐labeled pilot study of patients, reducing aura and headache symptoms in four of seven patients with otherwise intractable aura.

Two independent studies performed in humans report that amiloride is able to block the pain induced by application of acidic solutions under the skin (Ugawa et al. 2002) (Jones et al. 2004). These results are reinforced by the fact that the NSAIDs diclofenac and ibupropfen, which are also nonselective inhibitors of ASIC channels (Voilley et al. 2001), are able to attenuate acid‐evoked cutaneous pain in human volunteers without affecting the heat pain threshold (Jones et al. 2004).

A number of studies have presented evidence that ASIC1a activation also plays an important role in acidosis‐mediated neuronal injury (Gao et al. 2005; Pignataro et al. 2011). Sustained activation of these channels causes excessive influx of cations, such as Ca^2+^, Na^+^, and Zn^2+^, and leads to ischemic reperfusion brain injury (Leng et al. 2014).

However, the usual behavior of the channels is a rapid activation following a rapid acidification of the extracellular fluid followed by desensitization. The time constant of desensitization is ~1.2 sec for ASIC1a (Bassler et al. 2001) and ~0.5 sec for ASIC3 (Sutherland et al. 2001). Thus, it is not clear how the channels can mediate or amplify prolonged pain signals. In some cases ASICs carry “window currents” in a pH range over which channels are partially activated and incompletely desensitized. Such currents through ASIC3 are thought to play a role in the heart (Yagi et al. 2006). Similar window currents in cultured mouse DRG neurons were small (<5% of peak currents) but occurred at a pH of 7.2 which is within the range of many pathophysiological conditions. In other cases ASICs can mediate a persistent current that does not completely desensitize even at low pH. Examples include ASIC3, which can mediate persistent currents at pH <5.0 (Salinas et al. 2009), and shark ASIC1b which conducts sustained nonselective currents at pH <6.6 (Springauf and Grunder 2010). In our experiments, low pH induced persistent inward currents in cultured DRG neurons. However, these appeared to be mainly, if not entirely, due to other pathways, as they persisted when extracellular Na^+^ was completely replaced by NMDG^+^.

The clearest example of such an alternate pathway involves proton block of K^+^ channels. Since in most conditions K^+^ is accumulated in the cell against an electrochemical activity gradient, inhibition of outward K^+^ currents will depolarize the cell equivalent to activation of an inward current. These effects of low pH will be small at the cell resting potential but increase during depolarization (Fig. 5). In addition, some Cl^−^ channels, such as CLC2 and CLCK2, are activated by acidification of the extracellular fluid (Accardi and Picollo 2010). This will generate a depolarizing current at the cell resting potential resulting from outward movement of Cl^−^. We observed a decrease in persistent acid‐induced currents when the cell Cl^−^ was reduced, consistent with this idea. Finally, many cells have proton‐specific channels that could carry inward H^+^ currents at low pH (Cherny et al. 1995), although the best defined of these channels depend on membrane depolarization for activation (DeCoursey 2013). It is possible that the inward currents we observed in the absence of extracellular Na^+^ and electrochemical gradients for K^+^ and Cl^−^ are mediated in part by proton channels. However, the physiological significance of these currents in DRG cells remains undetermined.

Pharmacological agents can enhance acid‐induced persistent currents and even generate them at normal physiological pH. These agents include Zn^2+^ (Baron et al. 2001), FRFamide (Lingueglia et al. 2006), amiloride (Waldmann et al. 1997a; Yagi et al. 2006), and PcTx‐1 (Chen et al. 2006; Baron et al. 2013). Paradoxically the latter two compounds also block ASIC currents. Amiloride at high concentrations (>560 *μ*mol/L) opens homomeric ASIC3 channels and heteromeric ASIC3 +  ASIC1b channels at neutral pH, and also synergistically enhances the channel activation driven by mild acidosis (Waldmann et al. 1997a; Adams et al. 1999; Yagi et al. 2006; Li et al. 2011). It is not known if the sites of action for stimulatory and inhibitory effects are identical or distinct. However, there is evidence that the activating effect of amiloride involves binding to the acidic pocket and nonproton ligand‐sensing domain in ASIC3 channel rather than to the conducting pore (Yu et al. 2011; Baconguis et al. 2014). We found evidence for pH‐independent activation of inward currents by Zn^2+^ and amiloride in DRG neurons (Fig. 7).

The physiological significance of these responses is unclear, but they may have clinical implications regarding pharmacotherapy. Clinical interventions with amiloride as an ASIC blocker, such as in migraine pain management, may both inhibit transient currents and stimulate sustained currents depending on the dose. In addition, the potentiating effects of Zn^2+^ at micromolar concentrations suggest that levels of this ion in extracellular fluids could affect the sensation of H^+^ and the pharmacological actions of channel blockers. Further translational research studies are needed to explore the potential value of such therapeutic approaches.

## Conflict of Interest

None declared.
